# Pathomechanism of intervertebral disc degeneration

**DOI:** 10.1002/jsp2.1076

**Published:** 2020-02-13

**Authors:** Takeshi Oichi, Yuki Taniguchi, Yasushi Oshima, Sakae Tanaka, Taku Saito

**Affiliations:** ^1^ Sensory & Motor System Medicine, Faculty of Medicine The University of Tokyo Bunkyo‐ku Tokyo Japan; ^2^ Department of Orthopedic Surgery University of Maryland School of Medicine Baltimore Maryland

**Keywords:** epidemiology, genetic, intervertebral disc degeneration, prevalence, progenitors

## Abstract

Intervertebral disc degeneration (IDD) is the main contributor to low back pain, which is a leading cause of disability worldwide. Although substantial progress has been made in elucidating the molecular mechanisms of IDD, fundamental and long‐lasting treatments for IDD are still lacking. With increased understanding of the complex pathomechanism of IDD, alternative strategies for treating IDD can be discovered. A brief overview of the prevalence and epidemiologic risk factors of IDD is provided in this review, followed by the descriptions of anatomic, cellular, and molecular structure of the intervertebral disc as well as the molecular pathophysiology of IDD. Finally, the recent findings of intervertebral disc progenitors are reviewed and the future perspectives are discussed.

## Graphical abstract

Pathomechanism of intervertebral disc degeneration. Several risk factors including genetic factors as well as mechanical stress, trauma and smoking, attributed to the development of intervertebral disc degeneration. These biological and environmental factors induce the reduction of cell numbers and transformation of intervertebral disc cells through several molecular mechanisms, resulting in decreased production of extracellular matrix due to increased catabolic activity and decreased anabolic activities. Thereafter, the structured integrity of intervertebral disc is lost and intervertebral disc degeneration is further accelerated.

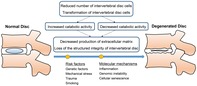

## 1 INTRODUCTION

Low back pain (LBP) is a common condition affecting approximately 637 million individuals worldwide.^1^ The high morbidity of LBP is associated with lower health‐related quality of life^2^ and high medical expenses,^3^ resulting in increased suffering and high socioeconomic costs. Intervertebral disc degeneration (IDD) is a major contributor to LBP,^4^ and it also precedes other spinal disorders such as disc herniation, spondylosis, and lumbar spinal stenosis.^5^ Herein, we will discuss the prevalence of and risk factors for IDD, structure of intervertebral disc (IVD), pathomechanism and histological features of IDD, and IVD progenitors.

## 2 PREVALENCE OF INTERVERTEBRAL DISC DEGENERATION

IDD develops during adolescence and progresses with age. In 1995, the prevalence of IDD, based on magnetic resonance imaging (MRI) findings, was described in a population‐based cohort in Finland.^6^ Among 232 men with a mean age of 49.3 (range, 35‐69) years, reduced signal intensity on MRI was observed in 41.6% and 86.0% of the participants at L1/2 and L5/S1, respectively. Reduced disc height was observed in 9.3% and 55.6% of the participants at L1/2 and L5/S1, respectively, suggesting that IDD is more frequent and severe at the lower lumbar disc than at the upper lumbar disc. In 2009, the prevalence of radiographic spondylosis was investigated in a large‐scale nationwide cohort study (Research on Osteoarthritis Against Disability; ROAD) performed in Japan.^7^ Among 2288 participants (818 men and 1470 women) aged ≥60 years, the prevalence of radiographic spondylosis with Kellgren‐Lawrence grade ≥2 was 75.8% in total, 84.1% in men, and 70.7% in women. Later, the prevalence of IDD based on MRI findings of the entire spine was reported in a population‐based cohort study in Japan,^8^ in which the presence of IDD was defined by Pfirrmann's grading system^9^ (where grade 4 and 5 indicated IDD). Among 975 participants (324 men and 651 women) aged 21 to 97 years, the prevalence of IDD was 71% in men and 77% in women aged <50 years, and >90% in both men and women aged >50 years. The prevalence of an intervertebral space with IDD was the highest at C5/6 (men: 51.5%, women: 46%), T6/7 (men: 32.4%, women: 37.7%), and L4/5 (men: 69.1%, women: 75.8%). LBP was associated with the presence of IDD in the lumbar region. Using the same cohort, Teraguchi et al. also examined the association between IDD and LBP, taking endplate signal change and/or Schmorl's node on MRI into consideration.^10^ Although IDD alone is not associated with the presence of LBP, the combination of IDD and endplate signal change was highly associated with the presence of LBP. More recently, the prevalence of Modic changes based on MRI findings of the lumbar spine was reported in a population‐based cohort study in China.^11^ Among 2449 participants with a mean age of 40.4 years, the prevalence of Modic changes was 5.8%, and the presence of Modic changes was significantly associated with the presence of IDD and correlated with the presence of LBP. Considering these results, endplate degeneration possibly plays an important role in the mechanism by which IDD causes LBP.

## 3 RISK FACTORS FOR IDD FROM EPIDEMIOLOGIC STUDIES

Before mid‐ to late 1990s, repetitive mechanical loading or wear and tear were believed to cause IDD. However, recent family and twin studies have suggested that the occurrence of IDD is determined largely by genetic factors, with environmental factors having an important role.^12^

### 3.1 Genetic factors

Genetic influences predominate among the reported risk factors for IDD. In 1995, a population‐based cohort study was performed using 115 male monozygotic twin pairs, in which IDD was evaluated by MRI.^13^ Familial aggregation explained 61% and 34% of IDD scores in the upper and lower lumbar spine, respectively, in multivariate analyses, suggesting that IDD is substantially affected by genetic factors and that compared with IDDs in the lower lumbar spines, those in the upper lumbar spines were more significantly influenced by genetic factors. The predominance of genetic factors was further confirmed by other twin studies, estimating that genetic factors account for up to three‐quarters of susceptibility to lumbar IDD.^14^, ^15^ Several candidate genes that may play a role in the onset of IDD have been reported by affected sib‐pair linkage studies or candidate‐gene association studies, such as *ACAN*,^16^ *CLIP*,^17^ *COL1A1*,^18^ *COL9A2*,^19^ *COL11A1*,^20^ *GDF5*,^21^ *IGF1R*,^22^ *IL‐1*,^23^ *IL‐6*,^24^ *MMP2*,^25^ *MMP3*,^26^ *MMP9*,^27^ *SKT*,^28^ *THBS2*,^29^ and *VDR*.^30^, ^31^ Further, recent genome‐wide association meta‐analyses identified novel candidate genes, including *PARK2* 32 and *CHST3*.^33^ An excellent review focusing on the extensive candidate genes was published recently.^34^ Many of these candidate genes are known to constitute the extracellular matrix (ECM) of IVD or be involved in ECM turnover, and, thus, they determine the size and mechanical property of the IVD by nature. Genetic defects in these genes presumably render the IVD more vulnerable against external force, leading to early onset of IDD. Further studies are required to elucidate the actual molecular mechanism through which each gene polymorphism causes IDD.

### 3.2 Mechanical stress

Excessive mechanical stress is thought to induce IDD, considering that IDD is more frequently observed in the lower lumbar spine, where IVDs suffer higher mechanical stress,^35^ and that IVDs adjacent to vertebral fusion are more likely to suffer IDD.^36^ On investigating a cohort of monozygotic twin pairs with different physical activities, Battie et al. found that physical activities explained only 2% to 7% of IDD scores in multivariate analyses.^13^ Similarly, in the aforementioned ROAD study, Muraki et al. examined the association between knee osteoarthritis/lumbar spondylosis and occupation/physical activity of the participants.^7^ Interestingly, the association between physical activity and lumbar spondylosis was weak, whereas the degree of physical activity was strongly associated with the presence of knee osteoarthritis, indicating that compared with knee joints, IVDs are less likely to be affected by mechanical stress.

### 3.3 Trauma

A study involving well‐matched cohorts, including 50 subjects who underwent discography and 52 control subjects, revealed that compared to matched controls, subjects who underwent discography showed accelerated IDD at 7 to 10 years of follow‐up; the progression of disc degeneration assessed by MRI was observed in 54 discs (35%) in the discography group compared to 21 discs (14%) in the control group.^37^ Further, retrospective clinical studies on 14 young patients with previous vertebral fracture and 14 healthy controls showed that IDD was more frequently observed in patients with previous vertebral fracture than in controls (57% and 8%, respectively).^38^ Hence, trauma is thought to be a risk factor for the onset of IDD.

### 3.4 Smoking

Smoking is the only chemical exposure known to be associated with the onset of IDD. On investigating the cohort of monozygotic twin pairs with different smoking exposures, Battie et al. found slightly greater IDD scores in the lumbar spine of smokers than in the lumbar spine of nonsmokers.^39^

## 4 STRUCTURE OF THE INTERVERTEBRAL DISC

**Figure 1:**
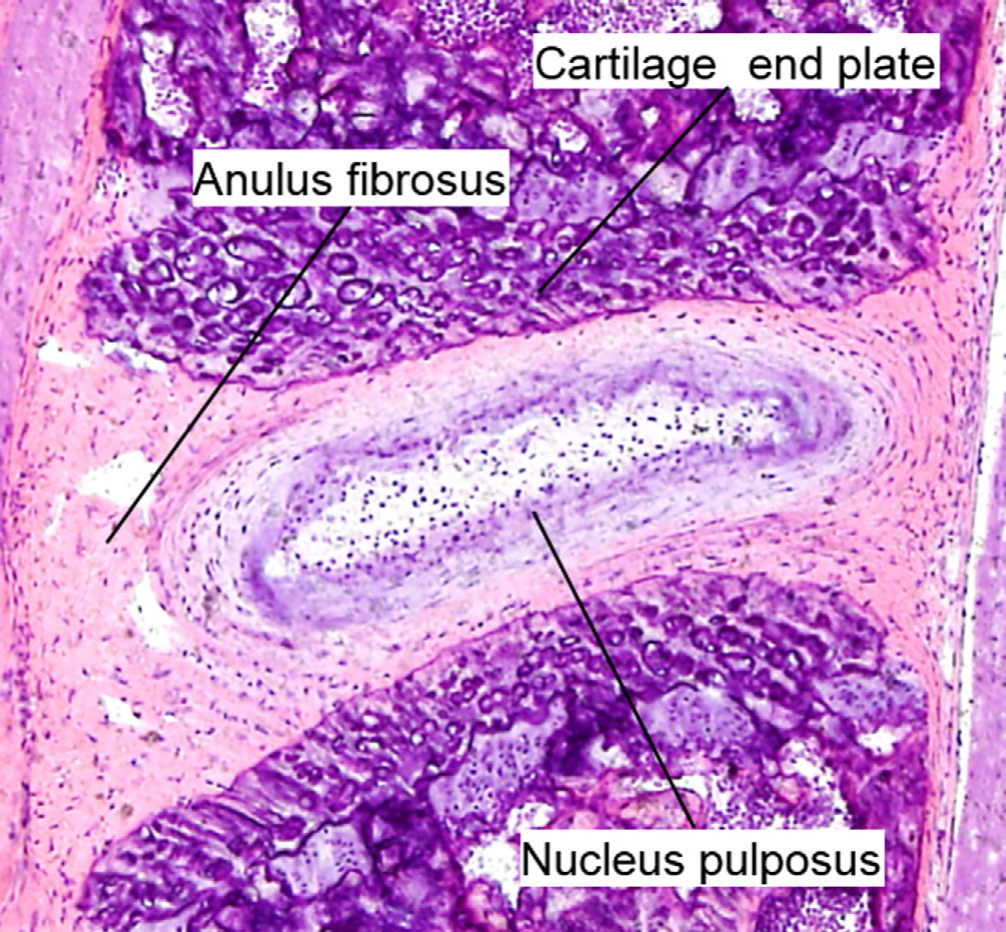
Hematoxylin and eosin staining of mouse lumbar intervertebral disc at 8 weeks of age

The IVD is composed of different but interrelated tissues, including the central highly hydrated gelatinous nucleus pulposus (NP), surrounding elastic and fibrous annulus fibrosus (AF), and cartilaginous end plates (CEP), which provides connection to the vertebral bodies (Figure 1).^40^ The NP is derived from the notochord, and notochordal cells remain in the tissue after birth and until around 10 years of age in humans. These cells are thereafter replaced by small chondrocyte‐like cells with lower metabolic activities.^41^ The ECM of NP consists of type II collagen fibers and elastin that contain proteoglycans such as aggrecan and versican. The presence of proteoglycans with negatively charged side chains makes the NP highly hydrated with high osmolarity, enabling the IVD to resist compressive loads and to deform reversibly.

The AF consists of a series of concentric rings, or lamellae, with collagen fibers lying parallel within each lamellae, providing tensile strength and the ability to withstand forces applied from any direction.^42^ The inner AF consists of several layers of fibrocartilage, while the outer AF is a fibrous tissue containing highly organized fibers composed mainly of type I collagen, allowing it to resist tensile loads.^43^ Proteoglycans and type II collagen fibers decrease gradually closer to the outer AF, while the content of type I collagen fibers increases.^44^

The CEP is a layer of hyaline cartilage that covers the caudal and cephalic ends of the disc, which plays an important role in the transport of fluids and solutes in/out of the disc.^45^ Similar to the articular cartilage, the ECM of the CEP consists of type II collagen embedded with chondrocytes.

## 5 PATHOMECHANISM AND HISTOLOGICAL FEATURES OF HUMAN IDD, AND LESSONS FROM ANIMAL MODELS

IDD can be attributed to several factors, including genetic factors as well as aging, mechanical stress, and injury. These biological and environmental factors induce the reduction of cell number and transformation of IVD cells, resulting in decreased production of ECM of IVD owing to increased catabolic activity and decreased anabolic activities. Thereafter, the structural integrity of IVD is lost and IDD is further accelerated.^46^, ^47^ One of the features of the pathomechanism of IDD in humans is increased catabolic and decreased anabolic activities, and the changes in ECM during the IDD process, characterized by the changes in the expression/structure of collagens/proteoglycans. Owing to the difficulty in obtaining human IVD samples, especially normal human tissue, several animal models that mimic these features have been developed to elucidate the pathomechanism of IDD.

### 5.1 Expression and structural changes of collagens

In humans, a general decrease in type II collagen production and a shift to type I collagen synthesis by NP cells or inner AF cells is observed as IDD progresses.^48^ In addition, localization of type X collagen has been observed in degenerated IVD, which is associated with the formation of cell clusters and clefts.^49^ During the process of development of IDD in humans, an increase in nonenzymatic glycosylation of collagen fibers is observed, leading to an accumulation of advanced glycation end‐products. As a result, cross‐linking of collagen fibers increases, causing tissue stiffness and rendering the IVDs more susceptible to mechanical damage during degeneration.^50^

### 5.2 Expression and structural changes in proteoglycans

Similar to the changes in the expression of collagens, a decrease in the proteoglycan content of human IVD is observed during degeneration.^51^, ^52^, ^53^, ^54^ In addition, the composition of glycosaminoglycan chains shifts from chondroitin sulfate to keratin sulfate,^52^ reducing the water content in the IVD. In synergy with the increased expression of type I collagen, the IVD becomes more fibrotic and less capable of withstanding mechanical stress.

### 5.3 Histological features of IDD

During the process of development of IDD in humans, several histological findings are observed in NP, including loss of demarcation between NP and AF owing to the shift of synthesis from type II collagen to type I collagen, dehydration caused by the decrease in proteoglycan production, presence of fissure, and cell cluster formation (Table 1).^55^ With regard to AF, disruption of the lamellar structure of collagen fibers, presence of fissure, and increased degree of vascularization and innervation are observed.^56^ Structural disorganization of the CEP is observed, including cracks, thinning, mineralization, microfracture in the adjacent subchondral bone, and bone sclerosis.^57^

**Table 1:** Histological findings of intervertebral disc degeneration

	Nucleus pulposus	Anulus fibrosus	Cartilage end plate
Changes at the molecular level	Decrease of proteoglycan Decrease of type II collagen Increase of type I collagen	Cross‐link of collagen fibers	Decrease of proteoglycan
Histological changes	Fissure Fibrosis Appearance of cell cluster Loss of notochordal cells and appearance of chondrocyte‐like cells	Disruption of lamella Fissure Vascularization and innervation	Microfracture and sclerosis of subchondral bone Thinning Reduction in the number of vascular channel
Biomechanical changes	Decrease of expansive force	Vulnerable against mechanical stress	

### 5.4 Pathological IDD and disc aging

Organismal aging results from time‐dependent accumulation of molecular and cellular damage, which leads to impaired tissue homeostasis, and eventual physiological and functional decline.^58^ Compared with other tissues, IVDs appear to undergo age‐related degenerative changes earlier in life.^35^, ^41^ On analyzing 44 human lumbar spines from deceased individuals without any spinal disorders, Boos et al. reported that age‐related histological changes of IVD include increased number and size of fissures, presence of granular debris, and neovascularization of the outer AF.^41^ On investigating 450 skeletons, Edelson et al. reported that the histological features of the CEP associated with aging include ossification and thinning of the CEP, microfractures in the subchondral bone, bone sclerosis, and reduction in the number of vascular channels in the CEP.^57^ As pathological IDD is caused by factors other than aging, such as genetic predisposition, trauma, and environmental factors, pathological IDD can occur in younger individuals and at a single intervertebral level, whereas disc aging is more systemic and is observed in older individuals in all spinal discs.^58^ However, we could not precisely distinguish between pathological IDD and IDD associated with aging owing to the almost similar histological features of the two conditions.^41^

With recent progress in aging research, some of the molecular pathways leading to disc aging have been elucidated. In particular, genomic instability and resulting cellular senescence have been determined to be important drivers of IDD.^58^ Each cell is constantly subjected to the risk of DNA damage due to the chemical instability of DNA structure, metabolic byproducts, and environmental mutagens and genotoxins.^59^ Despite several inherent DNA repair mechanisms of cells, the frequency of DNA damage becomes greater than that of DNA repair with aging, resulting in the accumulation of damaged DNA. It has been shown that accumulated genomic damage can lead to disc aging. For example, *Ercc1* ^*−/Δ*^ mice, in which ERCC1‐XPF is deficient (involved in DNA damage repair), showed typical features of disc aging, including loss of proteoglycan, decreased IVD height, and an increase in the number of senescent cells.^60^ The evidence that genotoxic stresses such as tobacco smoking or radiation cause disc aging further supports that DNA damage contributes to disc aging.^61^, ^62^ Other possible causes of DNA damage include oxidative stress induced by inflammation. Interleukin‐1 (IL‐1), a predominant cytokine involved in the pathogenesis of IDD,^63^, ^64^ has been demonstrated to induce cellular senescence in NP cells. Furthermore, IL‐1 receptor antagonist (IL1‐Ra) knockout mice showed typical features of human IDD, and NP cells from these mice showed the senescent phenotype.^65^

There are two types of cellular senescence, namely replicative senescence and stress‐induced premature senescence (SIPS). Replicative senescence is characterized by cessation of cell proliferation due to critical telomere shortening after consecutive replicative cell cycles.^66^ In contrast, SIPS is caused by the accumulation of genomic and mitochondrial damage. SIPS cells acquire a senescence‐associated secretory phenotype (SASP), which is characterized by the secretion of several inflammatory cytokines and matrix proteases that have profound catabolic effects on neighboring cells and ECM, promoting tissue degeneration.^67^, ^68^, ^69^ This pathomechanism of IDD is supported by previous studies that revealed that the number of senescent cells, which were assessed by senescence markers such as senescence‐associated β‐galactosidase and p16^INK4A^, were increased in human IVD samples. These markers were positively correlated with the expression of matrix metalloproteases including matrix metalloproteinase 13 (MMP13) and a disintegrin and metalloproteinase with thrombospondin motifs 5 (ADAMTS5).^70^, ^71^, ^72^, ^73^, ^74^ Recently, Patil et al. demonstrated the causal relationship between cellular senescence and age‐related IDD using the p16‐3MR transgenic mouse model in which p16‐positive senescent cells can be selectively eliminated by treatment with ganciclovir.^75^ The aging mice (age: 1 year) treated with ganciclovir showed decreased levels of catabolic factors along with improved histological features of IDD at age 2 years compared with control mice, indicating that cellular senescence has a direct impact on IDD development.

### 5.5 Lessons from animal models

Various kinds of inducers have been used to reproduce IDD in experimental animals, including compression,^76^, ^77^, ^78^ injury,^79^, ^80^, ^81^ instability,^82^, ^83^, ^84^, ^85^, ^86^ postural bipedality,^87^, ^88^ chemical,^89^ genetic,^90^ vibration,^91^ spontaneous,^92^ and smoking.^93^ Many of these models recapitulate the radiological and histological features of human IDD. Recently, we developed a mouse IDD model in which instability was induced without direct injury to IVDs, by surgical resection of posterior elements of the mouse lumbar spine.^94^ Radiological decrease in IVD height and histological findings compatible with human IDD were observed in this model. It is noteworthy that hypertrophic‐like chondrocytes appeared in the inner AF during degeneration, the morphologies of which apparently differed from those of normal inner AF, and these cells expressed type X collagen and MMP13,^94^ suggesting these cells contribute to IDD phenotypes as is the case in osteoarthritis.^95^ Thus, we speculate that the appearance of these cells in the inner AF may be a key event in the development of IDD. Further studies are warranted to investigate whether these cells are activated‐local progenitors or are recruited from other tissues, and whether prevention of these cells from expressing type collagen X and MMP13 is a potential intervention for preventing the progression of IDD.

## 6 INTERVERTEBRAL DISC PROGENITORS

The presence of local progenitors or the recruitment of appropriate cells into the damaged sites is required for tissue maintenance and repair.^96^ Despite preliminary results showing the positive effects of cell therapies in regeneration of the IVD, detailed basic research on IVD cells and their niche indicates that transplanted cells are unable to survive and adapt in the avascular niche of the IVD.^97^ It is imperative to identify the IVD progenitors and understand their niche to succeed in cell therapies for IDD. The IVD niche, which represents the unique microenvironment and communication network within the IVD cells, has been investigated by several researchers.^97^, ^98^, ^99^ The intervertebral disc is avascular because the capillaries terminate at the vertebral endplates and outermost AF, and the nutrition reaches the nucleus pulposus by diffusion through the CEP and outer AF.^100^, ^101^, ^102^, ^103^ As the NP, which is anatomically farthest from the vascular supply, is exposed to hypoxia, most energy for the NP is derived from anaerobic glycolysis.^104^ Anaerobic glycolysis in NP cells generates lactic acid and lowers the pH within the IVD. Other features of the IVD niche include low cellular density,^56^ high osmotic pressure,^105^ and high mechanical stress.^97^ IVD cells acquire specific adaptation mechanisms to survive in these harsh microenvironments.

### 6.1 IVD specific progenitors in vitro

Considering the harsh microenvironments in IVD, activation of endogenous progenitor cells could be a promising therapeutic strategy for IDD. In 2007, using an explant culture to isolate progenitors from degenerate human discs, Risbud et al. identified cells from both NP and AF, expressing typical marrow mesenchymal stem cell markers such as CD105, CD166, CD63, CD49a, CD90, CD73, p75 low‐affinity growth factor receptor, and CD133/1, and these results were also confirmed in rat IVDs.^106^ Thereafter, several researchers have reported the presence of cells compatible with MSCs from NP, AF, and CEP of normal or degenerated IVDs.^107^, ^108^, ^109^, ^110^, ^111^, ^112^, ^113^ Further, several microenvironments of IVDs, such as extracellular matrix stiffness, pH, and osmotic pressure, have been shown to affect the properties of these progenitors.^114^, ^115^, ^116^

Sakai et al. identified NP progenitors with novel NP‐specific cell markers, and demonstrated that these progenitors are exhausted with aging and degeneration.^117^ Using colony‐forming assay with methylcellulose semi‐solid medium, they identified progenitors from human and mouse NPs that express tyrosine‐protein kinase receptor (Tie2) and disialoganglioside 2 (GD2). These cells formed spheroid colonies that highly produced type II collagen and aggrecan and had multipotent and self‐renewal abilities both in vitro and in vivo. Tie2^+^GD2^−^ cells were found to be precursors of Tie2^+^GD2^+^ cells, and CD24, which was previously reported to be a specific marker of the NP,^118^ was found to be a specific marker of more mature NP cells, which differentiated from Tie2^+^GD2^+^ cells. Using these markers, NP cells were classified into four subtypes: dormant stem cells (Tie2^+^GD2^−^CD24^−^), self‐renewing stem cells (Tie2^+^GD2^+^CD24^−^), committed NP progenitor cells (Tie2^−^GD2^+^CD24^+^), and mature NP cells (Tie2^−^GD2^−^CD24^+^).^117^ Identification of these cell surface markers is epoch‐making in that it enables to evaluate the severity of IDD by quantifying the cell number and function, and in that it makes an index of induction of differentiation from other sources to become NP progenitor cells. Sakai et al. further revealed that angiopoietin‐1, a ligand of Tie2, suppressed apoptosis and promoted the proliferation of Tie2^+^ cells, enabling the development of a strategy to stimulate ANG‐1 to enhance Tie2^+^ progenitor cells for prevention of IDD.^117^ Later, the usefulness of the surface marker Tie2 was validated in a bovine coccygeal model.^119^

### 6.2 IVD‐specific progenitors in vivo

Some of the aforementioned progenitor cells maintain their potential multipotent differentiation and self‐renewal in vitro; however, knowledge regarding their in vivo characteristics, such as development, localization, or functional role in the maintenance of IVD homeostasis, is lacking.^97^

The methods to identify progenitors in vivo include label retention assay and lineage‐tracing experiments. In label retention assay, synthetic nucleic acid analogs, such as 5‐bromo‐2‐deoxyuridine (BrdU) or 5‐ethynyl‐2′‐deoxyuridine (EdU), are used to detect slow‐cycling cells, which are thought to be potential progenitor cells. A prolonged chase period results in dilution of the incorporated nucleic acid analogs, although the slow‐cycling cells remain labeled.^120^ In knee joints, slow‐cycling cells have been identified in the superficial zone, synovium, fat pad, top narrow reserve zone of the growth plate, and perichondrium (groove of Ranvier).^120^, ^121^, ^122^, ^123^ With regard to the IVD, Henriksson et al. identified slow‐cycling cells in a region close to the perichondrium, at the junction of the outer AF and the vertebral growth plate, suggesting the presence of stem cell niche.^124^ This region is analogous to the region known as the groove of Ranvier in the long bone.^122^ The same group proposed the migration routes of the progenitors from this stem cell niche to the outer AF by analyzing a cell adhesion and migration marker (β1 integrin), and EMT markers (Snail‐1 and ‐2).^125^

Lineage tracing provides more direct evidences, allowing us to follow cell fate decisions of progenitor cells and their descendants within a living organism without any perturbation.^126^ Lineage tracing typically uses the Cre‐loxP system to permanently mark the cells of interest. Cre recombinase is expressed under the control of a tissue‐ or cell‐specific promoter in one mouse line.^126^ That line is crossed with a second mouse line, in which a reporter is flanked by a *loxP‐STOP‐loxP* sequence.^126^ In animals expressing both constructs, Cre specifically activates the reporter in cells that express the promoter, by excising the *STOP* sequence.^126^ In the knee joint, several novel progenitors have recently been identified by lineage tracing. For example, Prg4‐creERT2 labels the progenitors of the superficial zone of the articular cartilage,^127^ PTHrP‐creERT2 labels the progenitors in the reserve zone of the growth plate,^128^ and Axin2‐creERT2 labels the progenitors in and around the groove of Ranvier.^129^ In studies on IVD, lineage tracing experiments by Choi et al. employing Shh‐cre and Shh‐creERT2 alleles showed that the notochord is the sole source of cells that form the entire NP.^130^ This was further confirmed by the lineage tracing experiments using the Noto‐cre allele.^131^ The AF and the CEP were devoid of Shh‐cre or Noto‐cre descendent cells, indicating that the progenitors of AF and CEP never reside in the NP. Although these studies provided important developmental findings, no creERT2 lines have been reported that can specifically mark putative progenitors in IVD tissues.

## 7 CONCLUSION AND FUTURE PERSPECTIVES

Recent population‐based cohort studies showed that IDD is very common and is associated with the presence of LBP and suggested that the degeneration of CEP has an important role in the pain associated with IDD. Previous epidemiologic studies have clearly shown that IDD is highly heritable. Although candidate gene approaches, family linkage analyses, and recent genome‐wide association studies have revealed several IDD‐sensitive genes, further studies are warranted to elucidate the molecular mechanisms through which each candidate gene causes IDD. The pathomechanisms of IDD have been elucidated using several animal models. However, caution should be exercised while interpreting the information obtained from animal models, as there are many differences between species, including disc size, cell type, nutrition, and mechanical forces.^132^ Several researchers have reported the presence of progenitors in IVD that behave like mesenchymal stem cells in vitro. However, the actual role and properties of the progenitors in vivo remain unknown owing to the lack of markers that can specifically mark the progenitors in vivo. Recent availability of single‐cell transcriptomic analyses possibly facilitates the identification of such marker genes that can track and localize potential progenitors.^133^ Establishment of the mouse line where Cre recombinase is expressed under the control of such marker genes will enable the purification of progenitors (eg, using fluorescence‐activated cell sorting), after knowing the properties of these cells in detail through comprehensive expression analyses such as RNA‐seq. Investigation of the signals that promote the function of progenitors will provide essential information for the development of drugs that can activate resident progenitors and, possibly, prevent IDD. In addition, identification of cell surface markers of IVD progenitors will contribute to not only the research of human IVD progenitors but also the development of exogenous cell therapies for IDD.

## CONFLICT OF INTEREST

The authors declare no conflicts of interest.

## AUTHOR CONTRIBUTIONS

T.O., Y.T., Y.O., S.T., and T.S. contributed the concept of the paper and wrote the manuscript. All authors have read and approved the final submitted manuscript.
